# Recent Studies on Kaposi’s Sarcoma-Associated Herpesvirus Circular RNAs

**DOI:** 10.3390/cancers17233743

**Published:** 2025-11-23

**Authors:** Cristian J. Pagtalunan, Isadora Zhang, Ariella Turley, Fenyong Liu

**Affiliations:** 1 School of Public Health, University of California, Berkeley, CA 94720, USA; 2 Department of Chemical and Biomolecular Engineering, University of California, Berkeley, CA 94720, USA; 3 Program in Comparative Biochemistry, University of California, Berkeley, CA 94720, USA; 4 Department of Chemistry, University of California, Berkeley, CA 94720, USA

**Keywords:** circular RNA (circRNA), herpesvirus, Kaposi’s sarcoma, Kaposi’s sarcoma associated herpesvirus (KSHV), non-coding RNAs, transcription

## Abstract

Kaposi’s sarcoma (KS), an AIDS-defining illness, is caused by Kaposi’s sarcoma-associated herpesvirus (KSHV). A member of the human herpesvirus family, designated as human herpesvirus 8 (HHV-8), KSHV is also linked to other oncogenic manifestations such as primary effusion lymphoma (PEL). The current dearth of available compounds against KSHV necessitates development of effective antiviral treatments. As with other herpesviruses, KSHV can result in both lytic and latent infections. KSHV pathogenesis and the development of KS have been associated with the expression of KSHV genes and transcripts during viral infections. The transcriptome of KSHV heavily intersects with regulatory pathways and mechanisms involved with a multitude of diseases in humans. Circular RNAs (circRNAs) have recently been discovered to be expressed by KSHV. Research endeavors on KSHV circRNAs have focused on the roles they play throughout latent and lytic infection. Understanding the specific functions and interactions of KSHV circRNAs with the viral and host transcriptomes, as well as how they are identified and analyzed, will be the primary focus of this review. Overall, recent advances in KSHV circRNA research have deepened our understanding of the KSHV transcriptome and pathogenesis and are paving the way for the development of circRNA-based antiviral therapies.

## Simple Summary

Kaposi’s sarcoma–associated herpesvirus (KSHV) causes cancers for which no vaccine exists, and current treatments are often imperfect. Recent work shows that the virus and infected cells produce circular RNAs, which are stable RNA loops that can influence gene activity and build up during active infection. Because these molecules resist degradation and may travel inside virus particles, they can help track disease or shape how the virus behaves. This review explains how viral circular RNAs are produced, how researchers detect and assay them, and what is known about their roles during quiet and active stages of infection in the context of KSHV. It also outlines experimental tools to test what these RNAs do and how to target them. By organizing current knowledge and practical methods, this review aims to guide future studies and speed progress toward better diagnostics and treatments.

## 1. Introduction

Kaposi’s sarcoma-associated herpesvirus (KSHV) [1], also known as human herpesvirus 8 (HHV-8), belongs to the γ-herpesvirus subgroup within the herpesvirus family [2]. It shares oncogenic properties with Epstein–Barr virus (EBV), another member of the herpesvirus family [3,4]. Understanding the KSHV life cycle and developing new strategies to treat this virus is crucial since no vaccine is currently available against KSHV infection [5]. Recently, virally encoded circular RNAs (circRNAs) have garnered increasing attention, and their roles in disease progression have become key areas of focus in research. Examining the specific capabilities of KSHV circRNAs and exploring how they are identified and analyzed will be the primary purpose of this review. We aim to summarize recent advances in circRNA research and discuss how these findings enhance our understanding of the KSHV transcriptome. By contributing to the knowledge surrounding KSHV circRNAs, we hope to facilitate efforts in developing antiviral circRNA treatments.

## 2. Kaposi’s Sarcoma-Associated Herpesvirus (KSHV)

KSHV is notoriously oncogenic, a characteristic shared with the cancer-causing human herpesvirus Epstein–Barr virus [4]. Since its discovery [1], KSHV has been directly linked to the pathogenesis of primary effusion lymphoma (PEL), multicentric Castleman’s disease, and serves as the causative agent of Kaposi’s sarcoma (KS)—a cancer that originates from mesenchymal cells and appears as reddish-brown lesions on the face, arms, and legs [2]. Transmission of the virus can occur through sexual contact, blood transfusions, and most commonly through salivary exchange [3]. These KSHV-associated malignancies often affect immunocompromised individuals, as the patient’s immune system is unable to control viral replication and manage the infection [2]. High-risk groups like sub-Saharan Africans, men who have sex with men (MSM), and HIV-infected individuals experience a higher prevalence of KSHV infection when compared to the general US population, often exceeding 30% in epidemiological studies [3].

Treatment plans are largely dependent on health factors unique to the patient, such as disease progression and symptom prevalence [6]. Recent advances made with devising treatment plans for KS have included patients who were also diagnosed with HIV. Highly active antiretroviral therapy (HAART) is utilized to induce immune reconstitution, but it was also found to be a potential defensive agent against the development of KS [2,7]. This primary treatment option, alongside surgical removal, immunotherapy, chemotherapy, and herpesvirus DNA polymerase inhibitors (i.e., foscarnet, cidofovir, ganciclovir, etc.), is considered a prominent method that can prevent progression of the disease, but these methods are often criticized for not being infallible [8].

KSHV possesses a double-stranded linear DNA genome of approximately 165 kb that encodes integral envelope, tegument, and capsid proteins necessary to form the virions [2,9]. The genome contains highly conserved open reading frames (ORFs) based on Herpesvirus saimiri (HVS) homologs, but some non-homologous sequences that are unique to KSHV are annotated consecutively with a “K” preceding the number [5,10]. The life cycle comprises two key infectious stages—the latent and lytic stages—each with a unique set of gene expression patterns [11]. During the KSHV latent stage, the virus maintains an expression pattern that features a few latent genes, such as ORF71/K13 (v-FLIP), ORF72 (v-Cyclin), ORF73 (LANA), and K12 (Kaposins) [12]. Other ORFs encode proteins that are responsible for the virus’s replication, structure, and transmission, including eight glycoproteins that provide virions access to potential hosts by complementary membrane fusion [13]. Successful membrane fusion and virion production require the virus to transition from the latent phase to the lytic phase, a process orchestrated by the coordinated activity of latent and lytic genes. [2].

The viral latency-associated nuclear antigen (LANA) plays a critical role in maintaining latency by suppressing the lytic infection stage [9]. LANA interacts with DNA methyltransferases to suppress genes that activate the lytic infection [14]. Under certain conditions, however, the virus can become reactivated and enter the lytic stage through cascading signals, thus starting the production and packaging of virions [9]. Multiple pathways are involved in promoting the transition from the latent stage to the lytic stage, including the activation of the protein kinase C (PKC) and mitogen-activated protein kinase (MAPK)/extracellular signal-regulated kinase (ERK) pathways [2]. Once these pathways are initiated, subsequent cascading signals permit the formation of the AP-1 complex, which is directly responsible for the expression of RTA, a key gene responsible for the KSHV lytic stage [15].

## 3. Host and Microbial circRNAs Found in Nature

Multiple forms of non-coding RNAs have been found to be transcribed throughout the KSHV lytic phase, promoting the virus’s regulatory functions. One novel class of RNAs produced by KSHV, known as circular RNAs (circRNAs) [16], is typically considered non-coding because they lack the 5′ cap structure and 3′ poly-A tail required to initiate translation. Previous ribosome profiling analyses have also suggested that most circRNAs are not associated with polysomes, although a few circRNAs have been shown to encode proteins [17,18].

CircRNAs produced by the KSHV genome have recently caught the attention of researchers due to their possible multifaceted capabilities [19,20]. The presence of circRNAs extends beyond viral genomes. Historically, a hallmark of circRNA research has been their discovery in many types of eukaryotic cells [21]. However, research focusing on viral circRNAs began when they were identified in the hepatitis delta virus (HDV) [16,22]. It is now recognized that circRNAs are nearly universal, with evidence of their expression across most organisms [23]. In other DNA viruses, such as Merkel Cell Polyomavirus (MCV), circRNAs and microRNAs (miRNAs) directly mediate the regulatory network that characterizes the viral transcriptome [24].

Generally, circRNAs are known to regulate gene expression, act as microRNA (miRNA) and protein sponges, serve as protein scaffolds, and have cancer-development expression patterns [21,22,25]. CircRNAs can act as molecular sponges by binding and sequestering miRNAs or regulatory proteins, thereby preventing them from interacting with their normal targets and altering downstream gene expression. Conversely, circRNAs can function as protein scaffolds by binding multiple regulatory proteins, bringing them into proximity to facilitate or modulate the formation of functional protein complexes. Their roles as miRNA and protein sponges are highly relevant to KSHV research, as many human circRNAs that are upregulated after infection are known to directly bind complementary miRNAs and affect subsequent protein function. Structurally, circRNAs are single-stranded molecules that form a closed circular loop.

Once thought to have been a benign byproduct of splicing during transcription [26], researchers have now begun discussing how circRNAs can become a potential therapeutic to treat diseases like those caused by KSHV [25]. By exploring circRNA mutagenesis techniques, lytic reactivation, and analyzing the subsequent RNA transcripts, researchers can characterize each circRNA within the KSHV genome, which can mobilize efforts to use these molecules as a means of treating infection.

## 4. Biogenesis of Viral circRNAs and Factors of Influence

**Figure 1:**
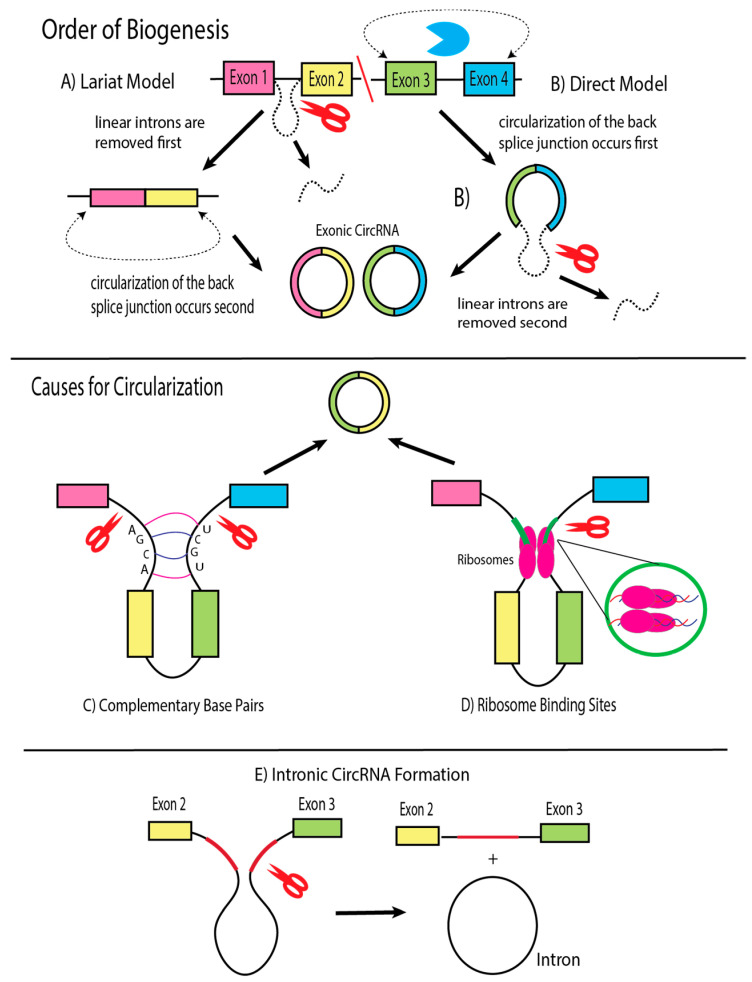
Summary of biogenesis factors that affect circularization of exons and introns in pre-mRNA transcripts. (**A**) The lariat model suggests that potential sites for circularization are first spliced to remove linear introns and then covalently connected into circRNA. (**B**) The direct model proposes circularization first before removing the intronic sequence. (**C**) The formation of circRNA via complementary sequences encourages circularization. (**D**) Certain sites flanking exonic sequences can be targets for RBPs, which facilitate circularization via splicing. (**E**) Exon-skipping splicing events can also occur, leaving circRNAs to consist of intronic sequences.

The biogenesis of KSHV-derived circRNAs follows mechanisms similar to those observed in cancer-related studies, particularly in primary effusion lymphoma (PEL) cell lines, where the vIRF4/K10 locus and PAN region exhibit high circRNA formation activity [19,20]. CircRNAs are generated by a non-canonical splicing event known as “back-splicing,” where a 3′ downstream end of a spliced exon is ligated to the 5′ end of the upstream portion of the exon, forming a phosphodiester bond [27] (Figure 1). The site at which the exon is ligated is referred to as the back-splice junction (BSJ). Discussion over the priority of canonical splicing and back-splicing events has resulted in two competing models: the lariat model and the direct model [28] (Figure 1). In the lariat model, canonical splicing first removes introns from the linear pre-mRNA, after which residual exons undergo back-splicing to form circRNAs. In contrast, the direct model proposes that back-splicing occurs prior to canonical splicing, generating circRNAs directly from precursor transcripts. In both models, residual introns are excised from immature circRNAs to yield mature circular molecules suitable for regulatory or translation functions [29]. However, exon-skipping events during splicing can lead to the generation of circRNAs containing intronic regions instead of purely exonic sequences.

Both models of circRNA biogenesis yield valid forms of circRNA. Certain factors, like *trans*-acting proteins and *cis*-regulatory elements, can additionally influence where RNA circularization may occur [21,30]. The four categories of *trans*-acting proteins include the spliceosome complex composed of small nuclear RNAs that form ribonucleoproteins (snRNPs), cleavage factors that remove the adjacent intron sequences, RNA helicases that unwind RNA to enhance the affinity for circularization, and RNA-binding proteins (RBPs) that mediate the distance between donor and acceptor sites to facilitate back-splicing [16,30]. In KSHV, ORF57 encodes a viral splicing factor that is involved with the splicing of several early lytic genes [31,32]. With regard to *cis*-regulatory elements, intronic complementary sequences, such as Alu repeats and polyadenylation sites (PAS), serve as splice sites for RBPs and cleavage factors to recognize [16]. Although the KSHV genome lacks Alu repeats—features specific to the human genome—the virus contains analogous flanking sequences that enable complementary base pairing during circularization (Figure 1) [33]. Computational analyses using the circRNA_finder, find_circ, and CIRI2 programs have shown that over 90% of KSHV circRNAs are flanked by short repeat or reverse-complementary sequences [21,34]. Another study utilized the PA-seq program to analyze results obtained from three separate KSHV-infected PEL lines to create a genomic landscape of all PAS [35]. The existence of PASs in pre-mRNAs directly hinders circRNA biogenesis as they promote binding to cleavage factors and reduce circRNA expression.

After successful transcription, most circRNAs remain intracellular. However, one study revealed the presence of viral circRNAs in purified KSHV virions, suggesting that viral circRNAs may be directed into the forming particles [27]. Unlike the linear counterpart, circRNAs are notorious for their resistance to exonucleases, which allows them to be exported to their corresponding destinations. Certain KSHV-encoded circRNAs, like circ-vIRF4, K7.3 circRNAs, and circPANs, were identified within the tegument of viral particles based on comparing samples treated with and without Triton X-100 [27]. However, viral circRNAs are not immune to complete degradation. Recent findings demonstrate that RNase L, an endoribonuclease activated during antiviral responses, plays a key role in degrading circRNAs expressed during viral infection, thereby contributing to their metabolic turnover [36].

## 5. Identification of KSHV circRNAs and Their Analysis by RNA-Seq Based Strategies

Bioinformatic approaches that analyze viral genomes within infected cells are central to characterizing circRNAs. Extensive studies have employed diverse computational algorithms to identify the origin of KSHV circRNAs [37]. In fact, the majority of KSHV circRNAs researched, and particularly the ones discussed in this review (Table 1), have all been identified or analyzed using these resources. Multiple cell lines have been utilized to study KSHV infection through RNA sequencing (RNA-Seq) [2,11]. By exploring circRNA expressions in multiple PEL cell lines, it was confirmed that identical circRNAs were universally present after reactivation of the KSHV lytic cycle [19,20,27]. In comparison, iSLK cells were derived from an HIV-negative SLK cell line and engineered to include a doxycycline-inducible gene, addressing issues related to the spontaneous reactivation of the lytic cycle [38]. This model allows for precise investigation of latency-associated RNA transcripts under controlled conditions. Additionally, studies using human umbilical vein endothelial cells (HUVECs) demonstrated that KSHV infection upregulates circRNA expression [20]. Because endothelial cells represent primary targets of KSHV infection in vivo, findings from these models are strongly validated by subsequent RNA-Seq analyses of the viral transcriptomes.

In terms of guiding factors that allow researchers to focus on a subset of circRNAs, interesting characteristics of potential candidates must reveal novel information. Certain research endeavors have focused on certain circRNA candidates due to their overall abundance with respect to transcript expression data [27]. Studies using algorithms to identify circRNAs that bind with complementary miRNAs also drive research endeavors to focus on understanding the interactome of circRNAs [20]. Potential candidates can even arise if they yield distinct expression patterns depending on the stage of infection.

Current research on relative changes in transcript expression has also shown that KSHV expression is conserved across multiple cell types. Related gammaherpesviruses such as EBV demonstrated evolutionary conservation of circRNA, as viral circRNAs were detected in rhesus macaque lymphocryptovirus-infected lymphomas from a rhesus macaque that was also infected with Simian Immunodeficiency Virus (SIV) [39]. Even though KSHV circRNA research that focuses on the evolutionary context for virally encoded circRNASs remains very limited, these previous attempts at exploring viral circRNA conservation demonstrate potential for future studies [39]. It should be known, however, that viral circRNAs are rarely ever conserved when compared to plant or animal circRNAs; only a few homologs between related viruses have been detected, but they do not concern the KSHV genome [40].

CircRNA profiles outlined by RNA-Seq offer insight into how mutant recombinant viruses influence circRNA transcript expression. One study focused on how the absence of viral interferon regulatory factor 1 (vIRF1) would affect RNA transcripts during lytic reactivation in EA.hy926 cells; by comparing volcano plots and heatmaps of circRNA transcripts between the wildtype and mutant viruses, they demonstrated reliable differentiation of circRNA expression [41]. The broader conclusion that viral factors affect circRNA expression was further established based on a recent RNA-Seq analysis on iSLK cells infected with a mutant version of KSHV that lacked circ-vIRF4 (Δcirc-vIRF4) [42]. By implementing qRT-PCR methods to analyze the RNAs isolated from vIRF1-transduced cells, scientists were able to identify certain circRNAs expressed from the human genome, thus directly establishing a connection between viral and human circRNAs [41].

From a computational perspective, several bioinformatic tools have been developed to improve the detection and analysis of viral circRNAs. Computational analyses of circRNAs often require prior enrichment with RNase R. Because circRNAs lack 5′ and 3′ ends, they are resistant to RNase R digestion, allowing linear transcripts to be removed and thereby improving the detection and sequencing of circular transcripts. One study implemented the CIRI2 program to analyze RNase R-treated RNA transcripts from a KSHV-infected BCBL1 cell line, and found that human herpesviruses contain highly conserved nucleotide signals surrounding the acceptor and donor sites [43]. Another paper describes using the CIRCExplorer3 and CLEAR pipeline programs [44] to map RNA-seq fragments to BSJ sites in order to evaluate the CIRCscore, a quantitative measure of how many reads directly span a junction. Their findings confirmed that circRNA expression was upregulated during lytic infection and further supported the involvement of circRNAs in immune signaling through the activation of Type I and Type II interferons (IFNs) [45].

**Table 1:** Summary of researched circRNAs expressed from both viral and human genomes during KSHV infection.

CircRNA	Location	Localization/Expression Level After Infection	Method of Analysis	KSHV or Human	Potential Functions and Oncogenic Phenotype	Reference
circvIRF4	K10 locus (vIRF4 gene)	Nucleus, cytoplasm, and detectable in patient sera; high level of abundance	RNA-Seq and ectopic expression	KSHV	Possible relation to oncogenesis	[42]
circPANs	PAN/K7.3 locus	High level of abundance	Divergent primers over BSJs BaseScope probing for transcript signals	KSHV	Potential biomarker for initial KSHV lytic activation in cancer cells	[27]
circK7.3						
kcirc3	ORF4, ORF6	Medium level of abundance	RNA-Seq detection of mapped reads and confirmation of BSJs; Used ectopic forms of circRNA expression from viral plasmids	KSHV	Unknown	[20]
kcirc29	K7/PAN	High level of abundance		KSHV	Unknown	
kcirc38	ORF21–22	Medium level of abundance		KSHV	Unknown	
kcirc54	ORF54	Low level of abundance		KSHV	Unknown	
kcirc55	ORF34–36			KSHV	Potential decoy for defense against antiviral human circRNA	
kcirc57	ORF34–37	Medium level of abundance		KSHV	Unknown	
kcirc97	ORF60–62			KSHV	Potential decoy for defense against antiviral human circRNA	
hsa_circ_0001400 (circRELL1)	RELL1	Cytoplasm; upregulated expressioon upon infection	RNA-Seq detection of mapped reads and confirmation of BSJs.	Human	Antiviral circRNA that dysregulates LANA and RTA	[20,46]
hsa_circ_0001741 (circTMPO3)	TNPO3	Unknown localization; upregulated expression upon infection	qRT-PCR analysis on RNA transcripts (Not conducted on KSHV-infected cell lines); RNA fluorescence in situ hybridization (FISH)	Human	miRNA sponge → regulatory roles in ovarian cancer and ESCC	[47]
hsa_circ_0005145	FAM105		Utilized varying neurological tissues, subsequently transfected and analyzed w/qRT-PCR (Not conducted on KSHV-infected cell lines)	Human	miRNA sponge → regulatory roles in mTLE	[48]
hsa_circ_0001808 (circARFGEF1)	ARFGEF1	Cytoplasm; upregulated expression upon infection	Comparison of reference in circBase; RT-PCR analysis on amplified regions via divergent primers	Human	miRNA sponge → facilitates pathogenesis of KSHV-related diseases	[41]

Many studies have used circRNA identifying algorithms based on potential BSJ-containing reads [49], but a recent article dedicated to implementing the computational framework, circRNA identification using A-tailing RNase R approach and Pseudo-reference alignment (CARP), revealed inconsistencies with just scouting for potential BSJ sites [50]. Similarly, other analyses have criticized popular pipelines for their dependence on specific RNA-Seq aligners to identify BSJ sites [51]. Contextualizing these limitations with regard to KSHV infection, additional reads with analysis purely based on BSJ sites may yield false positives, thus exaggerating the potential landscape of expressible viral circRNAs. Fortunately, updated algorithms, like CIRIquant [51], are able to remove false positives by refiltering the reads [49].

Additionally, short-read algorithms are often unable to characterize individual circRNA isoforms with precise exon compositions, but recent progress in long-read sequencing has shown potential in addressing this limitation. Long-read platforms such as PacBio and Oxford Nanopore can sequence entire circRNAs in single continuous reads, allowing researchers to determine their full exon structure and identify distinct isoforms that would otherwise be missed. For example, one study used isoCirc, a strategy that generates concatemeric constructs of long reads and aligns them during mapping to identify back-splice junctions (BSJs) and full-length isoforms, to produce a comprehensive catalog of circRNA isoforms across various cell lines [52]. Another protocol utilized CIRI-long, which combines rolling-circle reverse transcription with nanopore sequencing, to detect full-length circRNA isoforms at levels up to 20-fold higher than traditional Illumina-based methods [53]. Overall, these advancing technologies are helping to resolve the major challenges associated with relying solely on short-read sequencing.

## 6. KSHV circRNA Transcript Expression Patterns and Potential Functions for Therapeutic Means

So far, circRNAs expressed from the KSHV genome have garnered attention primarily for their association with biogenesis, lytic reactivation, and overall role as biomarkers [54]. Understanding their associations with other pathways can provide insight into their regulatory relationship with certain pathogenic mechanisms. Although current research has suggested direct involvement between circRNA transcripts and the expression level of latent and lytic genes, no causal conclusions have been made regarding the majority of the following KSHV circRNAs [55].

**Figure 2:**
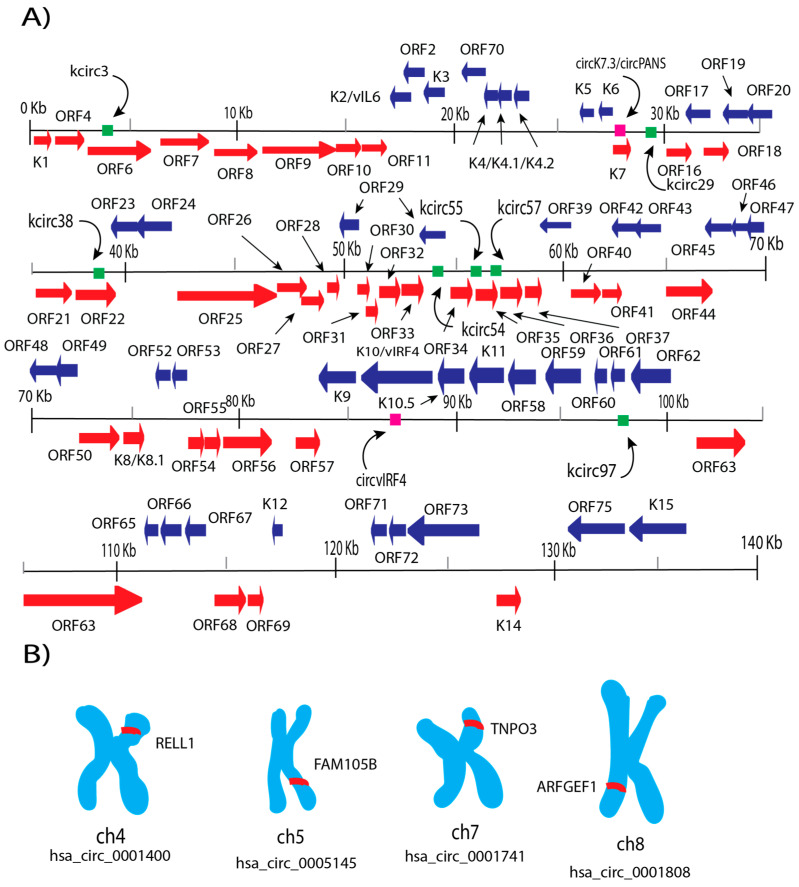
KSHV circRNA transcripts and four upregulated human circRNAs during KSHV infection. (**A**) Mapping of known KSHV ORFs alongside researched circRNAs that span certain loci in the KSHV genome [11,19,20]; ORFs are separated by color to distinguish direction of transcription. (**B**) Annotated chromosome locations of the upregulated human circRNAs and the genes in which they occupy [20,41,47,48,56].

Much of the research regarding expression level, conservation, and oncogenic phenotype of KSHV circRNAs is still being studied. Discovering the relationships between certain circRNA transcripts and the ORFs they overlap with (Figure 2) can reveal integral information about how these circRNAs are generated and their roles.

### 6.1. circvIRF4

One of the most researched circRNAs in the KSHV genome, circvIRF4 originates from the K10 genetic locus. It is considered to play a large role in regulating the cellular environment during the viral latent cycle [39]. The circRNA is generated by a BSJ that unites exons 1 and 2 of the vIRF4 gene.

Interestingly, circvIRF4 expression does not increase upon reactivation of latent KSHV across multiple cell types [39]. Several studies have discussed the dynamic interaction between circvIRF4 and the expression of other RNA transcripts. Using knockout cell lines, one study demonstrated that certain RNA-binding proteins (RBPs)—FUS, QKI, and RBM38—influence the balance between linear and circular transcript formation [57]. Another investigation revealed altered expression of extracellular matrix–associated proteins in circvIRF4 mutants. Particularly, an upregulation of SERPINE1 suggested involvement in the promotion of an angiogenic environment, which is representative of KS [42]. The detection of circvIRF4 in KS tumor samples further supports its potential role in viral oncogenesis, though additional studies are needed to define its expression patterns across KS and multicentric Castleman’s disease (MCD) tissues during tumor development [19].

### 6.2. circPANs

Another major locus analyzed in KSHV research is the polyadenylated nuclear (PAN) RNA locus. This region expresses over a hundred circRNA isoforms, which are collectively grouped and classified as circPANs [19]. The PAN locus is notable due to its predominant expression of linear PAN transcripts over circRNA; its nucleus-localized expression of linear transcripts suggests that accumulation is due to limited splicing and inefficient export to the cytoplasm [58]. These circRNAs are regulated by ORF57 and facilitate viral gene expression [59]. Unlike circvIRF4, there are considerable increases in the expression of circPANs immediately after cell lines were reactivated into the lytic cycle [55]. BaseScope analysis, which enables visualization of RNA transcript expression in situ, has demonstrated substantial expression of circPANs post-reactivation, suggesting a possible use of these circRNAs as a biomarker for tumorigenic lesions in KS [27]. Although studies have demonstrated that latency heavily drives the growth of KS lesions, there is evidence that suggests some lytic reactivation is required to initiate KS pathogenesis, solidifying the role that circPANs may serve as a biomarker [60]. The analysis uncovered more than a hundred unique RNA transcripts that were derived from the PAN/K7.3 locus in the nucleus and cytoplasm, which explains the relatively strong signal intensity compared to many other virally encoded circRNAs [27].

### 6.3. K7.3 circRNAs

Similar to circPANs, K7.3 circRNAs originate from the complementary antisense strand of the same locus that produces circPANs and demonstrate very similar characteristics [19]. The K7.3 circRNAs are consistently generated at a low frequency and span a variable range of nucleotides around the BSJ site [19]. Although numerous isoforms are produced, the overall abundance of each isoform remains low as back-splicing occurs at multiple positions. This apparent discrepancy may be explained by their subcellular localization and packaging into infectious virions prior to release from the host cell [27]. Ultimately, the increased expression of K7.3 circRNAs, alongside their complementary counterparts, circPANs, may play important roles in KSHV infection and pathogenesis, including tumorigenesis. However, defined functional roles relating to K7.3 circRNAs still remain limited [55].

### 6.4. ORF-Related circRNAs

Other circRNAs in KSHV that have undergone analysis are annotated by their placement in the genome and are denoted with the prefix “k” (Figure 2, Table 1) [20]. Given the current lack of research that focuses on their functions, most of these circRNAs have uncharacterized roles. Although they exhibit increased expression during lytic reactivation, this is likely attributable to their overlap with actively transcribed lytic genes, including *ORF4*, *ORF6*, *K7*, *PAN*, *ORF21*, *ORF22*, *ORF34–37*, and *ORF60–62* [20,27]. Specific analyses regarding kcirc55 and kcirc97 demonstrated a reduction in their expression in the presence of a human circRNA, hsa_circ_0001400, suggesting potential host defense mechanisms that sequester viral circRNAs through miRNA sponging or competitive interactions [20]. Otherwise, their transcription would directly correlate to the expression patterns of LANA and RTA in the absence of the antiviral human circRNA hsa_circ_0001400. One review describes a dose-dependent interaction with the expression levels of RTA, thereby validating the conclusion that the circRNA is involved with regulating KSHV infection [55].

### 6.5. Comparison Between KSHV circRNAs and Existing Biomarkers for KSHV Infections

These identified circRNAs present new opportunities for monitoring cancer progression associated with viral replication. Previous studies have examined the therapeutic relevance of circRNAs in clinical diagnoses of non-KSHV-related malignant cancers, particularly hematological malignancies; because of their resistance to exonuclease-mediated degradation, circRNAs are more stable than linear RNA transcripts and can persist longer in biological tissues, which may improve clinicians’ ability to monitor cancer progression [61]. However, the use of circRNAs as biomarkers in the context of KSHV infection requires a more complete understanding of the viral transcriptome and its downstream interactions with the host proteome. Continued investigation is necessary to determine how these circRNAs contribute to viral pathogenesis and whether they can serve as reliable diagnostic or prognostic indicators. In contrast to historically utilized KSHV biomarkers such as LANA and RTA, KSHV-derived circRNAs are detectable in patient sera. LANA and RTA are predominantly localized within the nucleus [62,63]. This extracellular stability suggests that circRNAs may serve as circulating biomarkers.

The reliable detection of KSHV circRNAs in serum has the potential to provide a less invasive alternative to tissue-based biopsies for monitoring viral infection and cancer progression. Although there is a lack of clinical application of KSHV-encoded circRNAs, research demonstrating their distinct detectability compared to conventional KSHV biomarkers provides a strong rationale for developing new diagnostic strategies that incorporate circRNA-based detection.

## 7. Upregulated Human circRNA Expression upon KSHV Infection

Investigating the interaction between KSHV and the human transcriptome has provided valuable insight into the regulatory pathways underlying numerous human diseases [2]. Although some of these circRNAs do not directly affect the KSHV lytic cycle, they are involved in other cancer tumorigenesis mechanisms due to regulatory interference with certain miRNAs (Figure 2, Table 1). Even outside the context of KSHV infection, human circRNAs are often found to directly affect cell growth and proliferation based on how host genes are expressed [16,64]. Their capacity to interfere with miRNA-based transcription is their unifying characteristic, but these mechanisms can either inhibit or exacerbate the potential for cancer. Additionally, the expression of these human circRNAs in response to KSHV infection is largely present in multiple patient samples, suggesting a robust conservation among humans [39]. However, like KSHV-encoded circRNAs, research that focuses on the expression of upregulated host circRNAs within primates remains limited. Microarray analyses have identified over 5000 human circRNAs transcribed during KSHV infection, providing researchers with extensive opportunities to investigate the roles of these circRNAs in KSHV infection and pathogenesis [20].

### 7.1. hsa_circ_0001400

Analysis of circRNA expression patterns during KSHV infection has identified a potential antiviral human circRNA, hsa_circ_0001400 (also known as circRELL1), which interferes with the expression of key latent and lytic viral genes [20]. This circRNA is upregulated upon infection in a variety of cell lines infected with other members of the human herpesvirus family, including EBV and human cytomegalovirus (HCMV) [43]. Essentially, the upregulation of this circRNA is functionally conserved across a multitude of herpesvirus infections [39,40]. Predicted mRNA targets of hsa_circ_0001400 were found to be enriched for transcription factors associated with chromatin remodeling [20]. As a result, the suggestion that human circRNAs can interfere with mechanisms that require certain RBPs further demonstrates their potential as “sponges” and overall antiviral capabilities. Protein interactions with hsa_circ_0001400 involve a PNN-interacting serine and arginine-rich protein (PNISR) that may play a direct role in the back-splicing of this human circRNA [46]. By increasing the driving force to circularize via back-splicing, PNISR decreases the amount of linear RELL1 transcripts [46]. Remarkably, exogenous expression of hsa_circ_0001400 led to a near-universal reduction in abundantly transcribed viral genes, underscoring its potential as a candidate for antiviral circRNA-based therapy.

### 7.2. hsa_circ_0001741

Human circRNA hsa_circ_0001741 has also been explored in the context of miRNA sponging and cancer proliferation outside of KSHV-related contexts [65]. This circRNA is upregulated during KSHV infection, and studies in ovarian cancer have shown that it inhibits tumorigenesis by targeting miR-188-5p, thereby enhancing the expression of FOXN2 [66]. However, this circRNA was also found to promote cancer proliferation in the context of esophageal squamous cell carcinoma (ESCC) by binding to miR-491-5p. When this miRNA could not bind to its target, NOTCH3, hsa_circ_0001741 promoted cell growth and division within esophageal squamous cells [47]. In other words, this human circRNA was found to be both oncogenic and non-oncogenic depending on the type of cancer. We should note that it is currently almost impossible to confirm any antiviral or tumor suppressive potential with relation to KSHV, as research regarding the involvement of hsa_circ_0001741 in viral pathogenesis remains limited.

### 7.3. hsa_circ_0005145

The human circRNA hsa_circ_0005145, whose expression is upregulated during KSHV infection, has been shown to sponge miRNAs that regulate IL-1α [48]. The target miRNA, hsa_miR-149-5p, was demonstrated to be associated with neuroinflammation in patients diagnosed with medial temporal lobe epilepsy (mTLE) [48]. Immunohistochemical and immunofluorescent studies have revealed that asymptomatic HIV-positive individuals can potentially harbor KSHV infection affecting cells within the central nervous system, including neurons and oligodendrocytes [67]. Although limited research has been conducted on this topic, these findings confirm that certain KSHV-induced host circRNAs are involved in other regulatory networks that do not pertain to KSHV viral replication. Specific effects on the KSHV transcriptome currently remain unknown.

### 7.4. hsa_circ_0001808

Alternatively known as circARFGEF1, the human circRNA hsa_circ_0001808 originates from the ARFGEF1 gene locus and is activated by vIRF4, a KSHV-encoded viral protein that interacts with the transcription factor, Lef1 [41]. As a result, a higher detection of circvIRF4 can indicate a subsequent increase in expression of circARFGEF1, but it should be known that circvIRF4 does not directly facilitate the increase in expression. CircARFGEF1 functions as a sponge to miR-125a-3p, a microRNA that targets GLRX3 and interferes with its function. Reports demonstrated that a knockdown of GLRX3 reduced cell proliferation set forth by proviral factors like vIRF1, which indicates that the presence of miR-125a-3p demonstrates antiviral properties. Therefore, the upregulation of circARFGEF1 would sequester the antiviral microRNA and encourage motility, proliferation, and angiogenesis in vivo [41]. As a result, this pro-growth circRNA exacerbates cancer progression, which makes it another potential target for therapeutics.

## 8. Research Regarding Exogenous Expression and Utilizing Knockouts to Study Functionality of KSHV circRNAs

Functional characterization of KSHV circRNA transcripts remains limited, as expression-based analyses primarily capture large fold changes following lytic reactivation without directly revealing molecular function. The application of the genome editing methods based on clustered regularly interspaced short palindromic repeats (CRISPR) and associated Cas proteins offers a promising approach for functional validation by enabling phenotypic comparisons between knockout and wild-type strains [68]. CRISPR-mediated mutagenesis relies on guide RNAs (gRNAs) that direct Cas endonucleases to complementary genomic loci, where they introduce double-stranded DNA breaks [68].

Research employing the CRISPR/Cas13 system has demonstrated its potential for eliminating expression of certain circRNAs. One study utilized the mutagenic technique to eliminate the expression of circGCN1L1, a circRNA found to sponge miRNAs (miR-330-3p) in patients who were diagnosed with temporomandibular joint osteoarthritis (TMJOA) [69]. Similarly, targeted deletion of circRNA loci in rice using single-guide RNAs (sgRNAs) flanking the circularization regions successfully generated circRNA knockouts, validating the system’s versatility across different genomes [70]. High-throughput CRISPR screening has also advanced circRNA research, as studies have constructed Cas13d libraries to systematically identify circRNAs and study their functional profiles [68]. However, there are drawbacks to using this technique, as it cannot be utilized in conjunction with large screenings for circRNAs. Since using traditional CRISPR-based mutagenesis techniques to form knockouts (KOs) will prevent linear versions of transcripts, researchers are limited to screening one KO at a time [71]. As a result, incorporating the previously mentioned high-screening method can allow for efficient analysis of key circRNAs.

It is noted that CRISPR is not the only mutagenesis technique for KSHV-upregulated circRNAs. Other techniques, such as mutagenized primers, are capable of activating lambda-red mediated homologous recombination in *E. coli* to create explicit knockout versions of a KSHV-containing BAC-mid, such as KSHV BAC16, the bacterial chromosome (BAC) that contains a KSHV genome [72,73]. Another strategy prioritizes base editors to allow for single-base nucleotide changes around genomic BSJs [71]. Certain analyses were able to conclude that imposing these mutations offered a reduction in corresponding circRNAs, but did not impair expression of identical linear transcripts [71]. Constructing knockout versions of a KSHV-containing BAC-mid using this method can provide dependable controls, as the only version of the transcript to be removed would be the circular version. In this manner, the linear transcript can provide its potential function, which will further establish that any differentiation post-reactivation will be due to the lack of the circRNA transcript.

One of the principal challenges in constructing circRNA knockouts is isoform complexity. To understand isoform behavior, one study demonstrated that one potential BSJ can result in multiple isoforms, thereby preventing researchers from focusing on a single isoform just by using mutagenesis techniques to remove the BSJ [74]. Other studies have acknowledged the difficulty in verifying any differences in circRNA transcripts due to isoform production and have proposed using differential primers to verify and distinguish isoform expression patterns [75]. Additionally, since the lytic ORF57 protein is associated with circRNA biogenesis and can affect isoform expression patterns, researchers may need to understand the mechanisms by which ORF57 drives transcription prior to studying certain circRNAs [32]. In the context of research surrounding KSHV circRNAs, these obstacles continue to prevent researchers from effectively studying upregulated circRNA transcripts upon infection. Ultimately, knockout-based experiments may be hindered by the inability to examine specific relationships to each individual circRNA expressed upon lytic infection.

## 9. Future Challenges and Direction

The research dedicated to KSHV circRNAs is rapidly growing and progressing, and a lot of potential circRNAs have not been characterized in detail. There are still many obstacles involved in studying the KSHV circRNA transcriptome, such as the limited tools to uncover the functionality behind viral circRNAs. Efforts in devising protocols for circRNA mutagenesis are needed to obtain reliable results. Future attempts that explore utilizing mutagenesis techniques in conjunction with circRNA knockouts can help drive research initiatives to understand the functions of these RNAs. By fully understanding the behavior and dynamics of certain circRNAs, scientists can potentially capitalize on their capabilities and develop antiviral molecular therapies for KSHV and herpesviruses in general.

## 10. Conclusions

KSHV causes several types of human cancer, including Kaposi’s sarcoma. CircRNAs encoded by KSHV have recently been discovered. Research on KSHV-derived circRNAs continues to illuminate their complex roles in viral replication, latency, and tumorigenesis. Their structural stability, regulatory versatility, and flexible means of biogenesis position them as promising biomarkers and potential therapeutic targets. Despite advancements in identifying and characterizing KSHV circRNAs, understanding their precise functional roles in viral infection and pathogenesis remains a significant challenge. Integrating RNA-Seq analyses, mutagenesis techniques, and virological studies will be crucial in uncovering their biological roles in supporting KSHV infection and causing KSHV-associated diseases. As studies progress, deciphering how KSHV circRNAs interact with both viral and host pathways may ultimately pave the way for novel antiviral strategies and improved treatments for KSHV-associated diseases.

## Data Availability

Not applicable.
